# Cheilitis Glandularis of Both Lips: Successful Treatment with a Combination of an Intralesional Steroid Injection and Tacrolimus Ointment

**DOI:** 10.1155/2018/9169208

**Published:** 2018-03-18

**Authors:** Norberto Sugaya, Dante Migliari

**Affiliations:** Division of Oral Medicine Clinic, Department of Stomatology, School of Dentistry, University of Sao Paulo, Sao Paulo, SP, Brazil

## Abstract

Cheilitis glandularis (CG) is an inflammatory condition of unknown cause that predominantly affects the minor salivary glands of the lips. Although a diagnosis of CG is not difficult, its treatment is a challenge. This article highlights the clinical presentation of the disease together with a case of successful management of this disease using a combination of a steroid injection followed by a topical immunosuppressor.

## 1. Introduction

Cheilitis glandularis (CG) is a rare inflammatory condition that predominantly affects the minor salivary glands and surrounding tissues of the lips. It affects adults (over 40 years old) to a greater extent than young people and almost exclusively white individuals. The ratio of male/gender involvement is 3 : 1. To date, no specific factor or cause has been associated with the disease onset [1].

The clinical features of CG lend to its diagnosis. The labial mucosa exhibits dilation of the orifices of the minor salivary glands through which thick saliva (mucin-rich) flows more intensely due to the inflammatory process inside the glandular parenchyma. This excessive salivary flow eventually dries out, leading to the development of yellowish plaques (or crusts) that cover the labial mucosa surface. These plaques are easily removed but form again, mainly during sleep. In addition to this main feature, the patient also develops enlargement and eversion of the lips. The vermilion border is typically not affected [2].

Although a diagnosis of CG is not difficult, its treatment is a challenge. The present article reports the successful management of a case treated with a combination of steroid injection followed by a topical immunosuppressor.

## 2. Case Report

**Figure 1:**
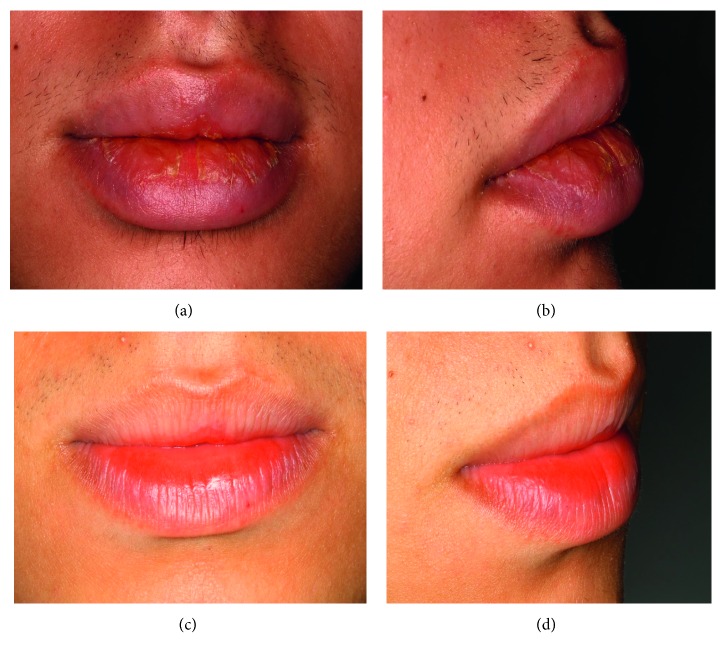
(a, b) Initial consultation with the patient exhibiting yellowish crusts on the labial mucosa. The lips (mainly the lower one) are also swollen and everted, whereas the vermilion border appears unaffected. (c, d) After treatment, both lips regained normal contour and aspect. A discreet redness of the lip mucosae remained unchanged throughout the follow-up.

A 16-year-old white male was referred to our oral medicine clinic for investigation of an enlargement of the lower and upper lips ongoing for approximately one year. His primary complaint was yellowish crusts on the mucosa surface of both lips but particularly the lower one (Figures 1(a) and 1(b)). Despite removal, the crusts reappeared mostly in the morning. He underwent previous treatments with topical corticosteroids to no avail. The patient was healthy with no history of substantive medical treatment.

Based on the aspect of the lesions, the diagnosis was cheilitis glandularis. The initial treatment was two intralesional injections of 10 mg triamcinolone suspension in both lips with a one-month interval between the applications. Two months following the second injection, an improvement was noticed with a reduction in the enlargement and eversion of the lips. Recurrent appearance of the crusts was also reduced but not to the extent that the patient felt comfortable. Instead of administering another steroid injection, a topical immunosuppressor (0.1% tacrolimus ointment) was applied twice daily for two weeks based on two effective management reports on CG found in the literature [3, 4] Before application, the patient was instructed to (1) wash his hands, (2) remove the labial crusts and apply 2% chlorhexidine gluconate on lip surfaces for disinfection, (3) allow the mucosa surfaces to dry and then apply the tacrolimus ointment, and (4) wash his hands again. This management procedure succeeded in completely resolving the lesions with no recurrence after a one-year follow-up (Figures 1(c) and 1(d)).

## 3. Discussion

CG is a rare disease but may occasionally be observed by the clinician. The lesion appears mainly through a process of renewed yellowish-plaque formation that is puzzling to health professionals. It seems that topical corticosteroids alone are not effective. Intralesional steroid injection has received some acceptance, but there is no consensus that it works in all cases. The mainstream treatment for CG has been vermilionectomy, but this treatment produces collateral effects, such as permanent itching and paresthesia.

Our patient did not require a biopsy. Biopsy was judged unnecessary because there was no clear-cut evidence that a biopsy would constructively help diagnose CG. Based on two relevant studies [1, 2], the histopathological findings were nonspecific, consisting mainly of chronic inflammation with various degrees of nonspecific sialadenitis and ductal ectasia of the minor salivary glands and fibrosis within the glands.

Possible differential diagnoses of CG may include contact cheilitis and cheilitis granulomatosa [1, 2, 5]. The first condition is attributed to irritants or allergic contactants. In these cases, apart from disclosing the contactant agent, the clinical features of contact cheilitis differ substantially from those of CG. For example, in the former, the lesions appear more prominent on the lip vermilion and are characterized mostly by the presence of adherent scales. Cheilitis granulomatosa shares some similarities to CG concerning lip swelling and eversion but lacks inflammation in the labial salivary glands, which leads to an increase of the salivary secretion and subsequent crust formation, a feature commonly observed in CG cases.

In the event of a difficulty in making a differential diagnosis between CG and cheilitis granulomatosa, a biopsy can be helpful as the latter can exhibit noncaseating granulomas; however, this feature is only observed in 40 to 50% of the cases. In either disease, the exact etiopathogenesis remains unknown. In a few cases of cheilitis granulomatosa, the development of this disease is potentially associated with food allergy (mainly cinnamon and benzoate compounds) [6].

Although one case of CG is not sufficient to judge the performance of a therapy, the treatment used in the present case appears to be effective. Tacrolimus was instrumental in preventing crust formation, possibly due to its effective action in controlling glandular inflammation by curbing the production of proinflammatory cytokines inside the minor salivary glands.
